# The Relationship Between Cholesterol Absorption and Intestinal Cholesterol Synthesis in the Diabetic Rat Model

**DOI:** 10.1155/EDR.2000.203

**Published:** 2000

**Authors:** Aine Gleeson, Daphne Owens, Patrick Collins, Alan Johnson, Gerald H. Tomkin

**Affiliations:** 1 Department of Clinical Medicine Trinity College Dublin Ireland; 2 Department of Biochemistry Royal College of Surgeons in Ireland Ireland; 3 The Adelaide and Meath Hospital Dublin Ireland

## Abstract

The chylomicron remnant particle is thought to be particularly atherogenic and we have previously shown alterations in post-prandial lipoproteins which could contribute to their atherogenicity. Cholesterol metabolism is disturbed in diabetes, yet the effect of diabetes on intestinal cholesterol synthesis and absorption has rarely been investigated. The aim of this study was to examine cholesterol absorption and intestinal synthesis of cholesterol in the streptozotocin diabetic rat. Twelve diabetic rats were paired with 12 control rats. [^14^C]-Cholesterol emulsion was administered and the lymph duct was canulated. Lymph was collected for 4 h. At sacrifice blood was taken for plasma lipoprotein measurements. Chylomicrons were prepared from the lymph by ultracentrifugation and [^14^C]-cholesterol content was determined by liquid scintillation counting. Lymph apolipoprotein B48 was isolated by gradient gel electrophoresis, and quantified by densitometric scanning. Serum triglyceride and cholesterol were greatly elevated in diabetic compared to control animals (260 ± 90 and 9.8 ± 8.0 mg/ml *vs.* 1.0 ± 0.4 and 0.6 ± 0.3 mg/ml, *p* < 0.0001 respectively). Lymph chylomicron apo B48 was similar in the two groups. Cholesterol absorption was not significantly different in diabetic compared to control rats but cholesterol synthesis was significantly, higher in the diabetic animals (550 ± 352 *vs.* 322 ± 113 *μ*g/h *p* < 0.03). There was a positive correlation between apo B48 and cholesterol absorption (*r* = 0.70, *p* < 0.01) in the diabetic rats and control rats (*r* = 0.71, *p* < 0.01) but no correlation between apo B48 and cholesterol synthesis in either group. This study demonstrates that cholesterol synthesis was increased in diabetes whereas cholesterol absorption was unaffected suggesting that intestinal cholesterol synthesis made an important contribution to the hypercholesterolaemia seen in the diabetic animals.


*Int. Jnl. Experimental Diab. Res.*, Vol. 1, pp. 203–210
Reprints available directly from the publisher
Photocopying permitted by license only

© 2000 OPA (Overseas Publishers Association) N.V.
Published by license under
the Harwood Academic Publishers imprint,
part of The Gordon and Breach Publishing Group.
Printed in Malaysia.

### The Relationship Between Cholesterol Absorption and Intestinal Cholesterol Synthesis in the Diabetic Rat Model

AINE GLEESON[a], DAPHNE OWENS[a], PATRICK COLLINS[b], ALAN JOHNSON[b] and GERALD H. TOMKIN[a,c,*]

[a]*Department of Clinical Medicine, Trinity College Dublin;* [b]*Department of Biochemistry, Royal College of Surgeons in Ireland;* [c]*The Adelaide and Meath Hospital, Dublin.*

*(Received in final form 14 December 1999)*

**The chylomicron remnant particle is thought to be particularly atherogenic and we have previously shown alterations in post-prandial lipoproteins which could contribute to their atherogenicity. Cholesterol metabolism is disturbed in diabetes, yet the effect of diabetes on intestinal cholesterol synthesis and absorption has rarely been investigated. The aim of this study was to examine cholesterol absorption and intestinal synthesis of cholesterol in the streptozotocin diabetic rat. Twelve diabetic rats were paired with 12 control rats. [$^{14}$C]-Cholesterol emulsion was administered and the lymph duct was canulated. Lymph was collected for 4 h. At sacrifice blood was taken for plasma lipoprotein measurements. Chylomicrons were prepared from the lymph by ultracentrifugation and [$^{14}$C]-cholesterol content was determined by liquid scintillation counting. Lymph apolipoprotein B48 was isolated by gradient gel electrophoresis, and quantified by densitometric scanning. Serum triglyceride and cholesterol were greatly elevated in diabetic compared to control animals (260 ± 90 and 9.8 ± 8.0 mg/ml *vs.* 1.0 ± 0.4 and 0.6 ± 0.3 mg/ml, $p < 0.0001$ respectively). Lymph chylomicron apo B48 was similar in the two groups. Cholesterol absorption was not significantly different in diabetic compared to control rats but cholesterol synthesis was significantly higher in the diabetic animals (550 ± 352 *vs.* 322 ± 113 μg/h $p < 0.03$). There was a positive correlation between apo B48 and cholesterol absorption ($r = 0.70$, $p < 0.01$) in the diabetic rats and control rats ($r = 0.71$, $p < 0.01$) but no correlation between apo B48 and cholesterol synthesis in either group. This study demonstrates that cholesterol synthesis was increased in diabetes whereas cholesterol absorption was unaffected suggesting that intestinal cholesterol synthesis made an important contribution to the hypercholesterolaemia seen in the diabetic animals.**

*Keywords*: Chylomicron; Apolipoprotein B48; Cholesterol synthesis; Cholesterol absorption; Diabetes

*Abbreviations*: MTP, microsomal triglyceride transfer protein; VLDL, very low density lipoprotein; LDL, low density lipoprotein; apo B48, apolipoprotein B48; ACAT, acylcoenzymeA cholesterol; acyltransferase HMGCoA, 3-hydroxy-3-methylglutaryl coenzyme A

### INTRODUCTION

The most important chronic complication of diabetes is atherosclerosis. It is generally

*Corresponding author. 1 Fitzwilliam Square, Dublin 2, Ireland. Tel.: 353 1 6761639, Fax: 353 1 6767122, e-mail: dowens@rcsi.ie accepted that disturbance in cholesterol metabolism is a major factor in the development of the atherosclerotic plaque. The role of hyperglycaemia is uncertain with strong correlation's between hyperglycaemia and atherosclerosis in many studies [1,2] while the benefit of reduction in blood sugar has yet to be conclusively demonstrated.[3,4] Reducing serum cholesterol has been shown to be beneficial in preventing myocardial infarction in non-diabetic subjects [5–7] while both hypercholesterolaemic [8] and normocholesterolaemic [6] diabetic patients showed even greater benefit from cholesterol lowering, in secondary prevention studies, than non-diabetic patients.

We and others have demonstrated alteration in intestinal function in diabetes.[9–13] In particular we have shown in the animal model, an increase in 3-hydroxy-3-methylglutaryl coenzyme A (HMGCoA) reductase and acylcoenzymeA cholesterol : acyltransferase (ACAT), [9,10] the rate limiting enzymes for cholesterol synthesis, and more recently we have demonstrated a 5-fold increase in microsomal triglyceride transfer protein (MTP) mRNA in the intestine of diabetic animals.[14] This enzyme is responsible for the assembly of the chylomicron.[15] These studies suggest an increase in synthesis of cholesterol, together with an explanation for an increase in the triglyceride load in the chylomicron in diabetes.[16] Although we have shown an increase in intestinally-derived apo B48-containing particles both in the chylomicron and the very low density lipoprotein (VLDL) fraction in diabetes,[16] we are uncertain as to the role of absorption and synthesis in relation to clearance with regard to these particles. Definition of the role of the intestine in cholesterol metabolism may have relevance to the targeting of pharmacological agents in the treatment of the dyslipidaemia of diabetes. Having described the increase in MTP mRNA in the intestine of diabetic animals we now report our investigations into cholesterol absorption as compared to intestinal synthesis. In this study we examine cholesterol absorption and synthesis in the streptozotocin diabetic rat model, isolating intestinal function by canulation of the lymphatic duct.

### Animals

Twenty four male Sprague Dawley rats weighing between 350 and 500 g were fed a commercial rat pelleted diet. The principals of laboratory animal care (NIH publication number 85.23) revised 1985 were followed. Animals were housed under licence from the Department of Health and experiments carried out according to Irish law as administered by the Department of Health. Twelve rats were made diabetic by subcutaneous injection with streptozotocin (Sigma, Poole UK) (60 mg/Kg in 0.2 mM sodium citrate buffer, pH 4). Each diabetic rat was paired with a control rat and the control rat was injected with buffer alone. Urinary glucose levels were monitored to confirm diabetic status. Rats with a urine glucose of $>24$ mmol/l were considered to be diabetic and diabetes was confirmed by blood glucose determination at sacrifice. The weight and food intake of each rat was monitored daily and the weight of the intestine measured at sacrifice.

### Lymph Duct Canulation

Five days after diabetes had been induced the diabetic and control rat pairs were switched to a diet of sunflower seeds. After two days on this diet, control and insulin-deficient rats were administered 1.5 ml of a freshly prepared [$^{14}$C]-cholesterol emulsion by gavage. The emulsion was prepared by mixing [$^{14}$C]-cholesterol (4.4 mCi) (Amersham, UK) and 110 mg non-radioactive cholesterol dissolved in chloroform, with 90mg phosphatidyl choline (Sigma Poole UK). The solvent was evaporated off under nitrogen and the mixture sonicated with warm (55°C) saline for 20 minutes using a Branson Sonifier 250 probe at 50% duty cycle, output 5 with a flat tip (Branson Ultrasonics, Danbury, CT, USA).

One hour after gavage rats were anaesthetised and a 2 mm portex tube inserted in the thoracic lymph duct through a transverse incision in the abdominal wall. The tubing was allowed to exit from the left hand side of the incision which was then closed with 4.0 nylon suture. A small section of the wound remained open to expose the stomach. Rats were hydrated hourly by gastric injection of 1.5 ml normal saline and lymph was collected for 4 hours. Rats remained anaesthetised for the entire procedure. After 4 hours rats were exsanguinated by cardiac puncture and the intestine removed and weighed.

### METHODS

### Lipoprotein Analysis

Triglyceride and cholesterol in rat serum was determined by enzymatic colorimetric methods using kits from Boehringer Mannheim (Mannheim Germany). Protein determination was by the Bradford method using Coomassie Protein Reagent (Pierce, Chester UK).

### Preparation of Lymph Chylomicrons

Lymph was collected for 4 hours immediately following canulation. Aprotinin (0.05 TIU) $NaN_3$ (0.02%, w/v) EDTA (0.1%, w/v), PPACK II (1 mM), and PMSF (0.1 mM) were added to protect the lymph from oxidation and degradation. The volume of lymph collected was measured and aliquots reserved for counting and analysis. The remainder was overlaid with 1.006 density solution and centrifuged at 10°C in a Sorval TFT 45.6 fixed angle rotor in a Sorval Combi Plus centrifuge (Dupont, Wilmington, DE, USA) for 30 minutes at 20,000 rev/min. The opaque layer at the top of the tube containing lymph chylomicrons was removed using a fine glass pipette, the volume was measured and chylomicrons were stored at 4°C. ApoB48 was determined within 1 week of lymph collection.

### Quantification of apo B48

Lymph apo B48 ($n = 9$) was measured using a modification of the method previously described for human plasma apo B48.[16, 17] Lymph chylomicrons were partially delipidated by mixing 62.5 µl of sample with 375 µl of diethyl ether. Samples were centrifuged for 15 minutes in microfuge, at 14,000 rev/min and the organic phase discarded. The protein was dried and redissolved in 24 µl of denaturing buffer (0.1 M Tris HCl, pH 6.8, 4% (w/v) SDS, 2% (v/v) β-mercaptoethanol, 0.01% (w/v) bromophenol blue, 20% (v/v) glycerol), heated for 5 minutes at 96°C, and loaded on a 4–15% polyacrylamide gel. Electrophoresis was for 1 hour at 60 mA constant current in 0.019 M Tris, 0.192 M glycine. LDL, isolated from human plasma by sequential ultracentrifugation was used as an apo B100 protein standard. Apo B100 protein standards (0.4 µg, 0.6 µg and 1.0 µg) were applied to each gel. LDL protein concentration was calculated from the mean value of ten determinations measured by modified Lowry procedure.[18]

Gels were stained for 1 hour with Coomassie Blue R250 0.1% (w/v) in methanol: water: acetic acid, (4 : 5 : 1 v/v) and destained with the same solvent. The bands were quantified by densitometry using Vilber Lourmat equipment (Vilber Lourmat Biotechnology, Marne le Vallee, France). Video images of the gels were generated and imported into Bio1D v6.32 software (Vilber) for analysis. Density values were assigned to the apo B100 bands of the human LDL and a standard curve constructed. Values were recalculated by linear regression and curves with a correlation coefficient 0.95 accepted. The concentration of apo B48 was determined from this standard. Previous researchers [19] demonstrated equal chromogenicity for apo B100 and apo B48 bands and we have shown that apo B100 staining is linear within the range 0.1–2.0 µg of protein. Inter and intraassay variation ($n = 6$) for apo B48 determination were 3.9% and 6% respectively.

### Cholesterol Absorption and Synthesis

Intestinal cholesterol absorption and synthesis were measured in the 12 pairs of diabetic and control rats. Lymph was collected for 4 hours and the volume measured.

To determine cholesterol absorption, [$^{14}$C]-cholesterol was measured in the emulsion and lymph by liquid scintillation counting using an LKB 1214 Rackbeta (Wallac). The specific activities of [$^{14}$C]-cholesterol was determined in emulsion and lymph. The amount of lymph [$^{14}$C]-cholesterol (cpm/4 h volume of lymph) was related to the specific activity of the [$^{14}$C]-cholesterol administered by gavage and divided by 4 to give μg cholesterol absorbed/h.

Cholesterol synthesis was determined by measuring total cholesterol in the 4 h collection of lymph, dividing by 4 to give the amount /h and subtracting the estimated amount of cholesterol absorbed/h

### Statistical Analysis

Results were expressed as mean ± standard deviation. Statistical analysis was performed using the Student paired *t*-test and ANOVA analysis of variance. Correlation coefficients were determined by linear and multiple regression analysis using Statworks software on an Apple Macintosh computer. Values of $p < 0.05$ were considered to be statistically significant.

### RESULTS

Rat characteristics on the day of test are shown in Table I. Mean weight of control rats at the beginning of the study was similar to the diabetic rats but at the end of the diabetic rats were significantly lighter than control animals ($p < 0.01$) demonstrating the usual weight loss of the diabetic animal model. Mean intestinal weight of control animals at the end of the study of was significantly lower than that of the diabetic animals ($p < 0.02$). Blood glucose for the control rats was $4.6 \pm 1.8$ *vs.* $17.2 \pm 3.4$ mmol/l for the diabetic animals ($p < 0.0001$).

Serum triglyceride in the control animals was $1.0 \pm 0.4$ mg/ml and in the diabetic animals $260 \pm 90$ mg/ml ($p < 0.0001$). Serum cholesterol was $0.6 \pm 0.3$mg/ml in control rats *vs.* $9.8 \pm 8.0$ mg/ml in the diabetic rats ($p < 0.001$). There was no difference in serum protein or lymph apo B48 between control and diabetic rats.

Cholesterol absorption was not significantly different in the diabetic compared to control rats ($20.2 \pm 19.4$ *vs.* 27.7 μg/h ± 27) (Fig. 1). Expressed as cholesterol absorption/g intestine, the diabetic animals having increased intestinal weight, it was also not different ($1.3 \pm 1.3$ μg/h/g intestine for diabetic rats *vs.* $2.0 \pm 1.9$ μg/h/g intestine for control rats). Cholesterol synthesis was significantly higher in the diabetic rats ($550 \pm 352$ *vs.* $322 \pm 113$ μg/h $p < 0.03$) and was still significantly greater when expressed per weight of rat intestine ($33.4 \pm 25.3$ *vs.* $22.4 \pm 8.4$ μg/h/g intestine, $p < 0.02$). Lymph apo B48 was measured in 9 rat pairs and was not significantly different between the two groups.

There was a positive correlation between lymph apo B48 and cholesterol absorption in the diabetic ($r = 0.70$, $p < 0.01$) and control rats ($r = 0.71$, $p < 0.01$) (Fig. 2) but no correlation between cholesterol synthesis and lymph apo B48 in either group.

TABLE I Rat characteristics on day of test

| | Diabetic | Control | $p <$ |
|---|---|---|---|
| Body weight | 368 ± 41 | 425 ± 61 | 0.01 |
| Intestinal weight (g) | 17.3 ± 2.7 | 14.7 ± 2.7 | 0.02 |
| Blood glucose (mmol/l) | 17.2 ± 3.4 | 4.6 ± 1.8 | 0.0001 |
| Serum triglyceride (mmol/l) | 260 ± 90 | 1.0 ± 0.4 | 0.0001 |
| Serum Cholesterol (mmol/l) | 9.8 ± 8.0 | 0.6 ± 0.3 | 0.001 |
| Lymph apo B48 (μg/h)($n = 9$) | 9.1 ± 4.0 | 11.3 ± 4.4 | ns |

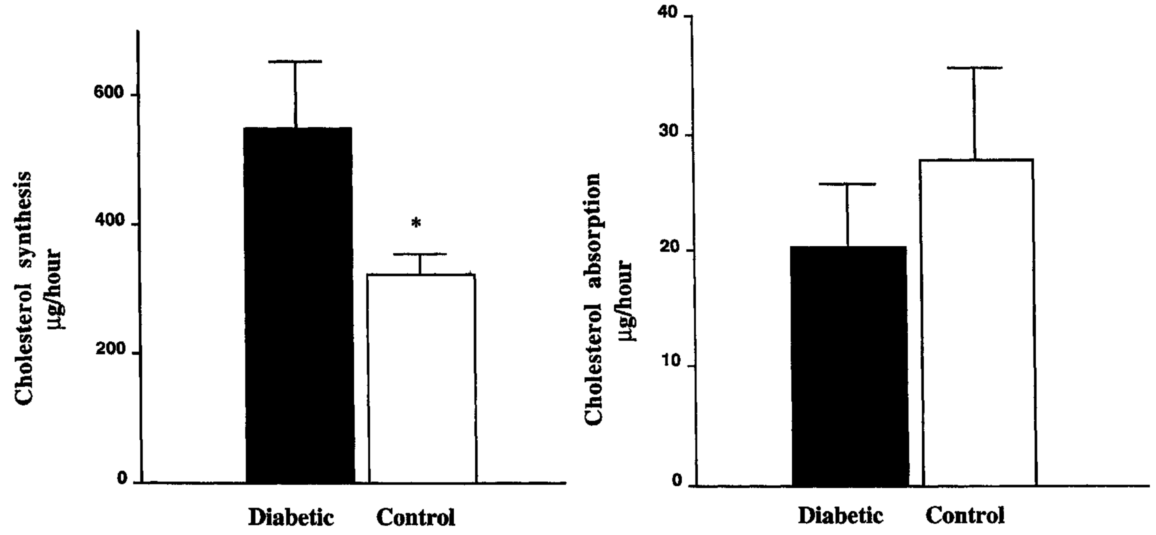

FIGURE 1 Cholesterol synthesis and cholesterol absorption measured after an oral [$^{14}$C]-cholesterol load in diabetic and control rat pairs. Chylomicrons were isolated from lymph by ultracentrifugation. Chylomicron [$^{14}$C]-cholesterol was related to the specific activity of the administered emulsion and to the amount of non-radioactive cholesterol in the chylomicron preparation. Mean ± sd. * $p < 0.05$ different from control.

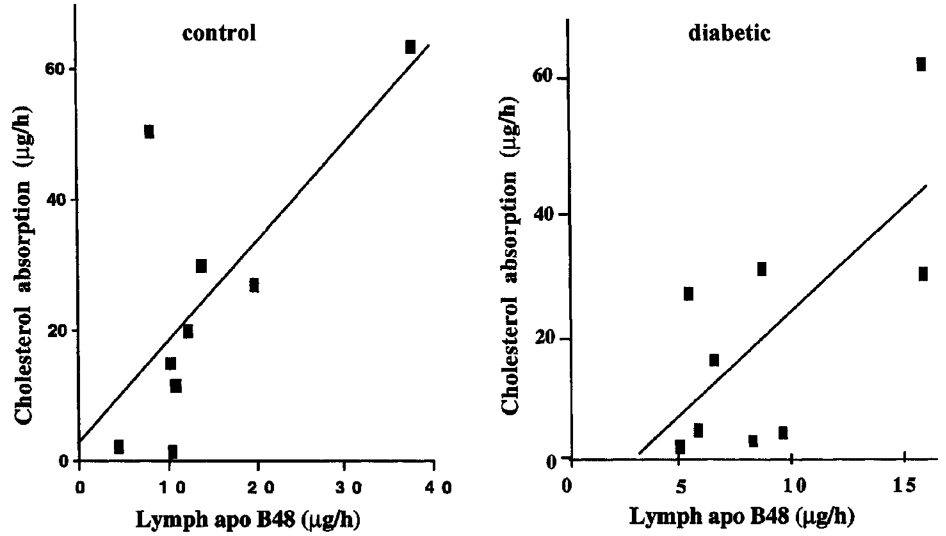

FIGURE 2 Relationship between lymph apo B48 and cholesterol absorption following an oral [$^{14}$C]-cholesterol load in diabetic and control rats. There was a significant positive correlation in both groups ($r = 0.71$, $p < 0.05$ for control rats and $r = 0.70$, $p < 0.05$ for diabetic rats).

### DISCUSSION

We have previously shown an increase in microsomal triglyceride transfer protein (MTP) in rat the diabetic intestine.[14] In that study lymph triglyceride and cholesterol were higher in the diabetic rats while apo B48 was similar. Diabetes in the rats was associated with weight loss hypercholesterolaemia and hypertriglycerideamia demonstrating insulinopaenia. Diabetes results in intestinal hypertrophy in animals and in this study we again found an increase in intestinal weight in the diabetic animals. We and others have previously demonstrated that the intestinal hypertrophy in diabetes is associated with an increase in HMGCoA reductase the rate limiting enzyme for cholesterol synthesis.[9–11]

We were particularly interested in this study in whether the hypertrophied intestine found in the diabetic rats would be associated with increased cholesterol absorption as well as increased cholesterol synthesis and the relationship between intestinally-derived cholesterol and apo B48. Our results demonstrate a significant positive relationship between lymph chylomicron apo B48 and cholesterol absorption demonstrating that cholesterol absorption in diabetes does not alter cholesterol content of the chylomicron particle. In spite of the increase in intestinal weight in diabetes, expression of results per g intestine did not alter the results significantly suggesting that the increase in synthesis is not solely due to intestinal weight in diabetes and this is supported by the finding of no increase in cholesterol absorption and indeed decrease in cholesterol absorption if defined by intestinal weight. On the other hand the increase in cholesterol synthesis in diabetes showed no relationship to apo B48 and was reflected in a cholesterol-enriched chylomicron particle. Strandberg *et al.*[20] had demonstrated low absorption and high synthesis of cholesterol and bile acids in normal subjects with high normal blood glucose with a suggestion that there was a relationship between insulin sensitivity and enhanced cholesterol synthesis. Increased cholesterol synthesis and decreased absorption of cholesterol have been reported in hypertriglyceridaemic subjects and more recently Gylling *et al.* [21] demonstrated, in type 2 diabetes, increased synthesis and decreased cholesterol absorption efficiency using non-cholesterol sterols to reflect absorption and synthesis. Our more direct measurements have also shown increased intestinal cholesterol synthesis but not absorption. This study allows us, for the first time, to separate absorption and synthesis from clearance. The method we have used to calculate absorption does not include cholesterol entering the intestinal mucosa from circulating lipoproteins, which is thought to be negligible in the postprandial state [22,23] however it is worth noting that serum cholesterol was more than 15-fold higher in the diabetic than control plasma. An interesting finding has been the very strong association between cholesterol absorption and apo B48 with no suggestion that intestinal cholesterol synthesis affects apo B48 production. Thus, a major defect of cholesterol metabolism in the intestine in diabetes is the production of a cholesterol-enriched particle.

Our recent finding of raised intestinal MTP mRNA in diabetes under similar experimental conditions[14] suggest that if MTP mRNA is translated into MTP protein, MTP does not stimulate apo B48 production. Van Greevenbroek *et al.*[24] found, in Caco-2 cells (an intestinally-derived cell line that secretes both apo B100 and apo B48) that MTP inhibition reduced apo B100 secretion but had no effect on apo B48. Thus in the intestine MTP appears to be important in regulating the triglyceride content of the chylomicron particle without increasing the number of particles. These studies demonstrate that in diabetes the increase in MTP mRNA is associated with a triglyceride-enriched particle.

The effect of particle size on plasma clearance of chylomicrons may be of great importance in our quest to understand the atherogenicity of the chylomicron particle. Martins *et al.*[25] have examined the clearance from plasma of chylomicron emulsion particles. When equal numbers of either small or large particles were injected into rats the large particles were significantly slower to clear than the small particles and larger particles were found to be lipolysed significantly less than small ones. It is possible that the delay in clearance of these large particles may impede the clearance of VLDL particles since apo B48-containing particles are preferentially taken up by the B/E receptor in the liver.[26]

In conclusion the present study has shown that in diabetes cholesterol absorption was not disturbed. Diabetes was associated with increased intestinal cholesterol synthesis but the increased cholesterol synthesis did not have an effect on apo B48 production. This may be the explanation for our previous finding of increased lipid/apoB48 in the newly formed chylomicron in diabetes.

### *Acknowledgments*

The authors would like to thank Dr. D. Guerrier and Ms. S. Laroche, Lipha Groupe, Centre de Research et Developpement de Lyon-Lacassagne for their financial support of the project. We are grateful to Dr. Kathy Botham London for teaching us the technique of lymphatic duct cannulation and to Ms. Phillipa Marks, Trinity College Dublin for carrying out the surgical procedures.

### *References*

[1] Coutinho, M., Wang, Y., Gerstein, H. C. and Yusuf, S. (1999). The relationship between glucose and incident cardiovascular events. A metaregression analysis of published data from 20 studies of 95,783 individuals followed for 12.4 years. *Diabetes Care*, **22**, 233-240.

[2] Turner, R. C., Millns, H., Neil, H. A. W., Stratton, I. M., Manley, S. E., Matthews, D. R. and Holman, R. R. (1998). Risk factors for coronary artery disease in non-insulin-dependent diabetes Mellitus: UK Prospective Diabetes Study (UKPDS: 23). *BMJ.*, **316**, 823-828.

[3] The Diabetes Control and Complications Trial Research Group (1993). Effect of intensive treatment of diabetes on the development and progression of long-term complications in insulin-dependent diabetes mellitus. *N. Engl. J. Med.*, **329**, 977-986.

[4] UK Prospective Diabetes Study (UKPDS) Group (1998). Intensive blood-glucose control with sulphonylureas or insulin compared with conventional treatment and risk of complications in patients with Type 2 diabetes (UKPDS 33). *Lancet*, **352**, 837-853.

[5] Shepherd, J., Cobbe, S. M., Ford, I., Isles, C. G., Lorimer, A. R., Macfarlane, P. W., McKillop, J. H. and Packard, C. J. (1995). The West of Scotland Coronary Prevention Group: Prevention of coronary heart disease with pravastatin in men with hypercholesterolaemia. *N. Engl. J. Med.*, **333**, 1301-1307.

[6] Lewis, S. J. *et al.* (1998). for the CARE Investigators. Effect of pravastatin on cardiovascular events in older patients with myocardial infarction and cholesterol levels in the average range. *Ann. Intern. Med.*, **129**, 681-689.

[7] The Scandinavian Simvastatin Survival Study (4S) Group. (1994). Randomised trial of cholesterol lowering in 4444 patients with coronary heart disease: The Scandinavian Simvastatin Survival Study (4S). *Lancet*, **344**, 1383-1389.

[8] Pyorala, K., Pedersen, T. R., Kjekshus, J., Fraegerman, A. G., Olsson, A. G. and Thorgeirsson, G. (1997). The Scandinavian Simvastatin Survival Study (4S) Group. Cholesterol lowering with simvastatin improves prognosis of diabetic patients with coronary heart disease. *Diabetes Care*, **20**, 614-620.

[9] Devery, R., O'Meara, N., Collins, P., Johnson, A., Scott, L. and Tomkin, G. H. (1987). Comparative study of the rate-limiting enzymes of cholesterol synthesis, esterification a catabolism in the alloxan-induced diabetic rat and rabbit. *Comp. Biochem. Physiol.*, **87b**, 697-702.

[10] O'Meara, N., Devery, R., Owens, D., Collins, P., Johnson, A. and Tomkin, G. H. (1990). Cholesterol metabolism in the alloxan-induced diabetic rabbit. *Diabetes*, **39**, 626-633.

[11] Feingold, K. R., Wilson, D. E., Wood, L. C., Kwong, L. H., Moser, A. H. and Grunfeld, C. (1994). Diabetes increases hepatic hydroxymethyl glutaryl coenzyme A reductase protein and mRNA levels in the small intestine. *Metabolism*, **43**, 450-454.

[12] O'Meara, N., Devery, R., Owens, D., Collins, P., Johnson, A. and Tomkin, G. H. (1991). Serum lipoproteins and cholesterol metabolism in two hypercholesterolaemic rabbit models. *Diabetologia*, **34**, 139-143.

[13] Martins, I. J., Sainsbury, A. J., Mamo, J. C. and Redgrave, T. G. (1994). Lipid and apoprotein B48 transport in mesenteric lymph and the effect of hyperphagia on clearance of chylomicron-like emulsions in insulin-deficient rats. *Diabetologia*, **37**, 238-246.

[14] Gleeson, A., Anderton, K., Owens, D., Bennett, A., Collins, P., Johnson, A., White, D. and Tomkin, G. H. (1999). The Role of microsomal triglyceride transfer protein and dietary cholesterol in chylomicron production in diabetes. *Diabetologia*, **42**, 944-948.

[15] White, D. A., Bennett, A. J., Billett, M. A. and Slater, A. M. (1998). The assembly of triacylglycerol-rich lipoproteins: an essential role for the microsomal triacylglycerol transfer protein. *Br. J. Nurt.*, **80**, 219-229.

[16] Taggart, C., Gibney, J., Owens, D., Collins, P., Johnson, A. and Tomkin, G. H. (1997). The role of dietary cholesterol in the regulation of post-prandial apolipoprotein B48 levels in diabetes. *Diabetic Medicine*, **14**, 1051-1058.

[17] Curtin, A., Deegan, P., Owens, D., Johnson, A., Collins, P. and Tomkin, G. H. (1996). *Elevated triglyceride-rich lipoproteins in diabetes: a study of apo B48 Acta Diabetologica*, **33**, 205-210.

[18] Markwell, M. A. K., Haas, S. M., Bieber, L. L. and Tolbert, N. E. (1978). A modification of the Lowry procedure to simplify protein determination in membrane and lipoprotein samples. *Anal. Biochem.*, **87**, 206–210.

[19] Karpe, F. and Hamsten, A. (1994). Determination of apolipoproteins B48 and B100 in triglyceride-rich lipoproteins by analytical SDS-PAGE. *J. Lipid. Res.*, **35**, 1311–1317.

[20] Strandberg, T. E., Salomaa, V. V., Vanhanen, H. T. and Miettinen, T. A. (1996). Association of fasting blood glucose with cholesterol absorption and synthesis in non-diabetic middle-aged men. *Diabetes*, **45**, 755–761.

[21] Gylling, H. and Miettinen, T. A. (1997). Cholesterol absorption, synthesis, and LDL metabolism in NIDDM. *Diabetes Care*, **20**, 90–95.

[22] Stange, E. F. and Dietschy, J. M. (1980). Cholesterol synthesis and low density lipoprotein uptake are regulated independently in rat small intestinal epithelium. *Proc. Natl. Acad. Sci. USA*, **80**, 5739–5743.

[23] Field, F. J., Born, E., Murthy, S. and Mathur, S. N. (1998). Caevolin is present in intestinal cells: role in cholesterol trafficking? *J. Lipid. Res.*, **39**, 1938–1950.

[24] Van Greevenbroek, M. M., Robertus-Teunissen, M. G., Erkelens, D. W. and de Bruin, W. A. (1998). Participation of the microsomal triglyceride transfer protein in lipoprotein assembly in Caco-2 cells: interaction with dietary saturated and unsaturated fatty acids. *J. Lipid. Res.*, **39**, 173–185.

[25] Martins, I. J., Mortimer, B.-C., Miller, J. and Redgrave, T. G. (1996). Effects of particle size and number on the plasma clearance of chylomicrons and remnants. *J. Lipid. Res.*, **37**, 2696–2705.

[26] Li, X., Catalina, F., Grundy, S. M. and Patel, S. (1996). Method to measure apolipoprotein B48 and B100 secretion rates in an individual mouse: evidence for a very rapid turnover of VLDL and preferential removal of B48 relative to B100-containing lipoproteins. *J. Lipid. Res.*, **37**, 210–220.

